# Attitudes of nursing staff towards computerisation: a case of two hospitals in Nairobi, Kenya

**DOI:** 10.1186/1472-6947-14-35

**Published:** 2014-04-29

**Authors:** Mathew K Kipturgo, Lucy W Kivuti-Bitok, Ann K Karani, Margaret M Muiva

**Affiliations:** 1 Kenya Medical Training College (KMTC), Nairobi, Kenya; 2 School of Nursing Sciences, University of Nairobi (UoN), Nairobi, Kenya

**Keywords:** Attitudes, Attitudes towards computerisation, Staff nurses, Computerisation

## Abstract

**Background:**

The health sector is faced with constant changes as new approaches to tackle illnesses are unveiled through research. Information, communication and technology have greatly transformed healthcare practice the world over. Nursing is continually exposed to a variety of changes. Variables including age, educational level, years worked in nursing, computer knowledge and experience have been found to influence the attitudes of nurses towards computerisation. The purpose of the study was to determine the attitudes of nurses towards the use of computers and the factors that influence these attitudes.

**Methods:**

This cross sectional descriptive study was conducted among staff nurses working at one public hospital (Kenyatta National Hospital, (KNH) and one private hospital (Aga Khan University Hospital (AKUH). A convenience sample of 200 nurses filled the questionnaires. Data was collected using the modified Nurses’ Attitudes Towards Computerisation (NATC) questionnaire.

**Results:**

Nurses had a favorable attitude towards computerisation. Non-users had a significantly higher attitude score compared to the users (p = 0.0274). Statistically significant associations were observed with age (p = 0.039), level of education (p = 0.025), duration of exposure to computers (p = 0.025) and attitudes towards computerisation.

**Conclusion:**

Generally, nurses have positive attitudes towards computerisation.

This information is important for the planning and implementation of computerisation in the hospital as suggested in other studies.

## Background

Contemporary healthcare faces a many changes on account of emerging and re-emerging diseases but nothing will change the way health care is provided more than the current advances in information, communication and technology. Nursing, being an integral part of the healthcare delivery system, is exposed continually to a variety of changes [1]. The responses of the nurses to change could vary from unreserved support to total rejection [2]. For instance, the introduction of computers can elicit diverse feelings among nurses. A positive attitude could see a rapid adoption with accompanying realisation of the benefits of computerization. A less positive attitude or rejection is likely to retard attempts to modernise service delivery.

Computers are among the many facets of information, communication and technology that have rendered the wide world a village [3]. In most parts of the globe, especially the developed nations, computers are used in almost all spheres of life [4]. Computers are ubiquitous accessories in all sectors of the economy, from the banking sector, transport, engineering, education, health sector etc.

In the 1990s the government of Japan gave incentives to hospitals willing to adopt electronic medical records (EMRs) [4]. Things are not different in the UK where Chan, Brew and Lusignan [5] reported that the health service had invested heavily in information technology. The establishment of a National Health Information System (HIS) in Canada has continued to receive government support through the Economic Action Plan [6]. In the USA, federal incentive payments for adoption and meaningful use of electronic health records (EHRs) are available for hospitals and office based physicians [7]. The payments could run into billions of dollars over a period of ten (10) years, according to the press release. And in Australia, the national and state governments were reportedly investing heavily in health information systems [8]. These efforts aim at mainstreaming information systems in the delivery of healthcare.

In Kenya, the use of computers in hospitals is not a widespread practice. Computerisation of hospital services has been embraced in some private (non-governmental) hospitals like the Aga Khan University Hospital and the Nairobi hospital. The other hospitals that have embraced computer systems include the Forces Memorial Hospital (a military run hospital), Nairobi Women’s Hospital and Metropolitan hospital. The extent of such computerisation is limited to non-clinical departments such as general administration, finance and procurement. Medical or health records department’s computerisation serves these non-clinical departments more than it does the clinical ones.

The minister of medical services Prof. Anyang’ Nyong’o [9] made a case for the adoption of information technology in the public health sector in his weekly column in a local newspaper. Should the minister follow up his call with action, the introduction of IT in Kenya’s public hospitals could happen sooner. Already efforts to adopt the use of computers in government owned hospitals have been made at Naivasha district hospital. In the African region, there is a dearth of published research on the computerisation of hospitals.

The Kenyan experience notwithstanding, the use of computers has slowly been implemented in many hospitals across the world [10]. According to Ragneskog and Gerdner [10], computers are no longer confined to hospitals but have been introduced in nursing homes and even in long-term facilities. Since nurses comprise the largest part of the health workforce, their acceptance of information and technology systems will be mandatory for implementation of ICT [8].

The purpose of this study was to document and compare the attitudes of nurses towards computerisation among nurses with and without computer experience and examine the factors influencing their attitudes.

### Literature review

#### Nurses attitudes towards computerisation

Concerning the attitudes of nurses towards computerisation, the literature is almost equally divided between those which found nurses to have positive attitudes and those which found them to have negative attitudes. A number of published studies have focused on the attitudes of nurses toward computerisation [11,12]. Most of these researches were carried out in the UK, USA, Australia, Taiwan and other developed countries. Literature search on the African continent revealed very little. This implied that either no research was conducted on this area or any that was carried out had not been documented. Consequently, the findings from these studies (from outside Kenya) may not necessarily be applicable in Kenya. The only documented studies were done by Kivuti-Bitok [13] and Kivuti and Chepchirchir [14] both conducted at KNH. Both studies focused on the nurse managers at KNH who were found to possess positive attitudes towards computerisation.

According to Fishbein and Ajzen (1975, _**p.340**_) quoted by Jayasuriya & Caputi [15] ‘attitude is a learned predisposition to respond in a consistently favourable or unfavourable manner with respect to a given subject’. The attitude of an individual can foretell his or her intention to perform a behaviour regarding an object of interest [15]. According to Stronge and Brodt (1985), nurses’ computer attitudes reflected their complex internal states that affect their choice or behaviour towards computer use.

Effectively, the attitudes of nurses towards computerisation will determine the success or failure of the program being introduced.

Other studies on the attitudes of nurses toward computerisation have detailed a variety of findings [16-19]. Getty et al. [20] compared the attitudes of users and non-users and found the non-users with previous computer experience to be of a more positive attitude. Lee [21] adapted Vroom’s expectancy theory and found a significant relation between nurses’ attitudes, satisfaction with computers and motivation to use computers while Shoham and Gonen [22] found a correlation between the work environment and attitude towards computers. In other studies, Ragneskog and Gerdner [10] and McBride and Nagle [17] compared the attitudes of nursing students and registered nurses towards use of computers in practice, found that the registered nurses believed students nurses were less competent in IT skills, while the two groups faced different challenges. Eley et al. [8], also state that nurses generally demonstrated positive attitudes towards computer use and acknowledged the benefits of ICT to clinical care. Their assertion is supported by Hwang and Park [23] who found that nurses had favorable attitudes toward computerisation.

Other studies have reported negative attitudes of nurses towards computers [24]. Sultana [25] found that nurses had negative attitudes towards computerisation.

Other authors brought to light the negative views held by nurses and reported that many had reservations with being exposed to a computer [26].

Reports from other studies like those carried out by Brumini et al. [19], Ahn et al. [27], Alquraini et al. [24], Lee et al. [28], Kivuti-Bitok [13] and Kivuti & Chepchirchir [14] indicate that nurses had favourable attitudes towards ICT. This suggests that over time, in this increasingly computerised world, the negative attitudes have been replaced by more positive ones.

### Factors that influence nurses attitudes towards use of computers

Several factors have been found to shape the attitudes of nurses towards the use of computer systems. Age, educational level, years of nursing experience and experience with computers have frequently been described as factors influencing nurses’ attitudes towards computerisation. According to Lee, et al. [28] cited by Huryk [6], age was consistently found to influence nurses’ attitudes towards computerised nursing care plans. Younger nurses demonstrated a greater enthusiasm in the use of the technology. Simpson and Kenrick [12] were in agreement when they asserted that younger, less experienced nurses had positive attitudes towards computerisation.

In an earlier study Brodt and Stronge (1986) discovered that the level of education, type of nursing and years of experience in nursing were associated with a positive attitude toward computers. Scarpa et al. [29], studying the attitudes of nurses in a non-computerized hospital, also found that previous experience with computers was an important contributor to a positive attitude. Similarly, Brumini et al. [19] established that computer education and experience were significant factors that contributed to the development of nurses’ positive attitudes towards computers. Correspondingly, following a comprehensive review of the literature, Laurie Huryk [6] found that computer experience was by far the most influential factor to nurses’ positive attitudes. Conversely, Sultana [25] reported no major relationship between attitudes and computer experience or any demographic variables.

### Rationale

It is a common practice for authorities to plan and institute changes in hospitals without regard for the feelings or views of the intended end users. Nurses frequently find themselves being compelled to adopt changes for which they had little or no contributions. This invariably breeds resentment and resistance leading to slowed or unsuccessful realisation of the change. According to Timmons [2] the responses of nurses to change could change from unreserved support to varying degrees of rejection. Numerous studies that have been conducted on the attitudes of nurses towards computerisation have been inconclusive.

Given the pace at which technology is spreading across the globe, the push for the adoption of computer technology in the provision of healthcare is likely to find many public hospitals completely unprepared. It is therefore necessary to understand the feelings of nurses towards computerisation, identify factors influencing those feeling and develop strategies for addressing undesirable factors that would hinder successful implementation.

### Research questions

The research questions for the study were based on questions arising from the literature review:

What attitudes do nurses with or without computer experience have towards computerisation?

Is there a difference in the attitudes of nurses with computer experience and those without computer experience?

What is the association between nurses’ age, education, years of nursing experience, experience with computers and their attitudes towards computerisation?

## Methods

### Population and sample

This was a cross sectional descriptive study to determine the attitudes of nurses towards the use of computers in two hospitals. Respondents from the two hospitals were purposively sampled. One was a private computerised hospital while the second was a public (non-computerised) Hospital. The population of the study included staff nurses employed at the two hospitals. The sample included 156 (78% of the nurses at KNH) and 44 (88% of nurses at AKUH). All the staff nurses who were working during the study period were included in the study. Participants from AKUH were designated users (because AKUH had adopted use of computers while those from the second hospital (KNH) were designated as non-users because KNH had not adopted use of computers. The respondents were either registered or enrolled nurses. Enrolled nurses are certificate holders, having trained for two and half years, while the registered nurses were diploma holders who spent three and half years in training.

### Inclusion and exclusion criteria

The inclusion and exclusion criteria for the study were as follows: Must be qualified nurses.

All staff nurses on duty who agreed to participate. Nurses on leave and student nurses were excluded.

Clinical nurse managers, supervisors and nurse specialists were also excluded. This was because some Nurse Managers, supervisors and some nurse specialists at KNH had some rudimentary/minimal access to computers at KNH.

### Data collection instruments

A modified version of the Nurses’ Attitudes Towards Computerisation (NATC) developed by Stronge and Brodt (1985) was used. The instrument comprises three sections: − demographic data, attitude statements and computer use questions. The questionnaire is attached.

### Data analysis

The data were entered and analysed using SPSS. Differences and associations between groups and variables were calculated using non-parametric tests (Kruskal-Wallis and Mann–Whitney U tests).

### Ethical considerations

A written approval for the execution of the research was received from the relevant research and ethics committees (University of Nairobi/Kenyatta National Hospital Ethics Review Committee). Full disclosure was made to the participants and their anonymity assured. The participants’ completion and return of the questionnaire was taken to mean consent for inclusion in the study.

## Results

### Demographic characteristics

Seventy eight percent (n = 156) of all the participating nurses were from KNH and the remaining 22 % (n = 44) were from the AKUH. Table 1 shows that the mean age of nurses in KNH was 36.7 years and 29.8 years at AKUH. Most (34%, n = 68) of the nurses were aged between 31 to 35 years of age. Seventy-one percent (n = 142) of all nurses in the study were female.

**Table 1:** Age distribution of nurses at KNH and AKUH

	**KNH**	**AKUH**	**Total**
	**Frequency (%)**	**Frequency (%)**	**Frequency (%)**
**Age**			
20-25 years	8 (5.1)	14 (31.8)	22 (11)
26-30 years	17 (10.9)	11 (25.0)	28 (14)
31-35 years	56 (35.9)	12 (27.3)	68 (34)
36-40 years	31 (19.9)	4 (9.1)	35 (17.5)
41-45 years	24 (15.4)	2 (4.6)	26 (13)
46-50 years	8 (5.1)	-	8 (4)
Over 50 years	12 (7.7)	1 (2.3)	13 (6.3)
Total	156 (100)	44 (100)	200 (100)

Most of the nurses within both hospitals (50.6% in KNH and 54.6% in AKUH) had diploma level nursing qualifications.

Over forty percent (40.9%) of the nurses in AKUH had durations of nursing practice ranging between 1–5 years while in KNH most 28.9% nurses had worked for 16 years and above (Table 2).

**Table 2:** Length of nursing practice among respondents

	**KNH**	**AKUH**	**Total**
	**Frequency (%)**	**Frequency (%)**	**Frequency (%)**
**Length of nursing experience**			
Less than 1 year	5 (3.2)	7 (15.9)	12 (6)
1-5 years	25 (16.0)	18 (40.9)	43 (21.5)
6-10 years	40 (25.6)	8 (18.2)	48 (24)
11-15 years	41 (26.3)	7 (15.9)	48 (24)
16 years and above	45 (28.9)	4 (9.1)	49 (24.5)

Ninety three percent (n =41) of the nurses in AKUH reported that they had experience in using computers compared to 64.1% (n = 100) of nurses at KNH (Table 3). More than half (54.6%, n = 24) of the nurses at AKUH had access to a computer at work compared to only one (0.6%) nurse at KNH. The majority (36.4%) of nurses at AKUH had used computers for durations of between 3 to 4 years, while in KNH most (20.8%) nurses had used computers for 1 to 2 years. There were missing responses at KNH (5.3%).

**Table 3:** Experience, access and use of computers among respondents

	**KNH**	**AKUH**	**Total**
	**Frequency (%)**	**Frequency (%)**	**Frequency (%)**
**Experience with computers**			
Yes	100 (64.1)	41 (93.18)	141 (70.5)
No	56 (35.9)	3 (6.82)	59 (29.5)
**Duration of computer use**			
Less than 1 year	28 (18)	7 (15.9)	35 (17.5)
1-2 years	32 (20.5)	14 (31.8)	46 (23)
3-4 years	28 (18)	16 (36.4)	44 (22)
5-10 years	13 (8.3)	4 (9.1)	17 (8.5)
Over 10 years	5 (3.2)	1 (2.3)	6 (3)
**Access to a computer**			
Work only	1 (0.6)	24 (54.6)	25 (12.5)
Home only	90 (57.7)	1 (2.3)	91 (45.5)
Both work and home	12 (7.7)	18 (40.9)	30 (15)
None	45 (28.9)	1 (2.3)	46 (23)

### Computer knowledge and practices

Nurses at AKUH scored higher on all the responses on computer knowledge and practice compared to their colleagues at KNH. Forty-one (93.2%) of the nurses in AKUH were able to operate computers compared to 48.1% of KNH nurses. Over three-quarters or 77.3% (n = 34) of nurses in AKUH reported that they were good at using a word processor compared to less than half 46.2% (n = 72) of nurses at KNH. In terms of use of information technology, all except one nurse in AKUH (97.7%, n = 43) had an email account. In KNH 68% (n = 106) of nurses indicated they had an email account. In addition, 63.6% of AKUH nurses reported that they were capable of preparing and using PowerPoint presentation while 48.7% of KNH nurses reported that they were similarly capable of using PowerPoint. Twenty-nine (61.4%) nurses in AKUH used computers on a daily basis compared to 23.7% of those at KNH (Table 4).

**Table 4:** Computer knowledge and practice among the respondents

	**KNH**	**AKUH**
	**Frequency (%)**	**Frequency (%)**
Ability to operate computers	75 (48.1)	41 (93.2)
Use computer mouse for navigation	112 (71.8)	43 (97.7)
Know how to use the key board for typing	109 (69.9)	42 (95.5)
Good in using the word processor	72 (46.2)	34 (77.3)
Conversant with computer vocabulary	77 (49.4)	33 (75)
Have an e-mail address	106 (68)	43 (97.7)
Capable of preparing and using Power Point presentation	76 (48.7)	28 (63.6)
Received formal computer training	73 (46.8)	29 (65.9)
Frequency of using computer		
Use the computer every day	37 (23.7)	27 (61.4)
Use the computer at least once in a week	65 (41.7)	25 (56.8)
Use the computer once in a month	27 (17.3)	10 (22.7)
Rarely use the computer	65 (41.7)	6 (13.6)

### Attitude of nurses towards computerisation

Table 5 indicates responses to individual attitude items. Attitude scores were calculated for each respondent by adding up all responses to the 20 items to yield a score with a possible minimum value of 20 and maximum value of 100. A higher score represented a more positive attitude towards computerisation. The total attitude scores for nurses at both hospitals was 73.53 (SD = 13.15) out of a maximum possible score of 100. The range of attitude scores for this sample was 34 to 100. Generally the nurses had a positive attitude towards computerisation. Nurses in KNH had significantly higher attitude scores (mean = 74.56) compared to those in AKUH (mean = 69.86, p = 0.0274). In response to individual attitude statements, most respondents strongly disagreed with the suggestions that computerisation could increase nursing workload (43.9% for KNH nurses), represented a violation of patient rights (42.8% for KNH nurses), will cause nurses to give less time to quality nursing (53.5% KNH nurses), increase workload (55.8% AKUH nurses), should never be used outside finance department (73.9% for KNH and 72.1% for AKUH nurses) and if possible nurses should never use computers (72.4% for KNH and 80.5% for AKUH nurses).

**Table 5:** Nurses’ responses to individual attitude items

	**KNH (%)**					**AKUH (%)**				
**Attitude statements**	**SA**	**A**	**U**	**D**	**SD**	**SA**	**A**	**U**	**D**	**SD**
A computer increases costs by increasing the nurses workload	5.2	11	4.5	35.5	43.9	16.3	Nil	2.3	23.3	55.8
Costs of health care are likely to increase because of computers	8.4	18.8	4.6	34.4	33.8	18.2	4.6	29.6	34.1	2.3
The time spent using a computer is out of proportion to the benefits	5.9	9.2	9.9	32.2	42.8	7.3	2.4	36.6	41.5	13.6
Computers represent a violation of patient privacy	6.7	17.3	8	31.3	36.7	17.1	14.6	22	34.2	12.2
Only one person at a time can use a computer terminal and, therefore, staff efficiency is inhibited.	16.2	27.9	10.4	26.6	18.2	27.3	9.1	15.9	20.5	12.2
Computerization of nursing data offers nurses a remarkable opportunity to improve patient care	49.7	35.5	2.6	5.2	6.5	40.9	29.6	22.7	6.8	27.3
Computers contain too much personal data to be used in an area as open as a nursing station	14.7	27.6	5.1	30.1	22.4	11.6	20.9	4.7	44.2	18.6
Computers can cause nurses to give less time to quality nursing care	8.3	11.5	6.4	35.3	38.5	14	14	4.7	14	53.5
If I had my way, nurses would never have to use computers	4	4.6	4	15.1	72.4	4.7	4.7	2.3	16.3	72.1
Computers should only be used in the financial department	4.6	0.7	2.6	17.7	73.9	4.9	2.4	2.4	9.8	80.5
Computers make nurses jobs easier	51.7	31.1	6	7.3	4	60.5	25.6	7	4.7	2.3
Paperwork for nurses can be reduced greatly by the use of computers	69.9	20.9	5.2	1.3	2.6	56.8	29.6	2.3	9.1	2.3
Orientation for new employees takes longer because of computers	7.1	8.4	11.7	38.3	34.4	4.7	11.6	9.3	37.2	37.2
Nursing data cannot be manipulated using computers	13.6	23.9	9.7	27.1	25.8	12.2	26.8	4.9	36.6	19.5
Computers save steps and allow the nursing staff to become more efficient	43.5	35.7	6.5	10.4	3.9	47.7	29.6	2.3	13.6	6.8
The more computers in an institution, the less number of jobs for employees	21.8	25	10.9	24.4	18	29.6	22.7	6.8	20.5	20.5
Increased computer use will allow nurses more time to give patient care	45.3	26	9.3	14	5.3	37.8	29.7	8.1	16.2	8.1
Because of computers, nurses will face more law suits	9.5	15.5	12.2	29.1	33.8	3	15.2	9.1	36.4	36.4
Computers can cause a decrease in communication between hospital departments	7.3	11.3	6.7	28.7	46	16.7	16.7	Nil	30.6	36.1
Confidentiality will be sacrificed by patient records being computerized	14	17.3	6	33.3	29.3	32.4	27	2.7	18.9	18.9

Key: SA = Strongly Agree; A = Agree; U = Undecided; D = Disagree; SD = Strongly Disagree.

A significant percentage of nurses in AKUH were undecided on whether time spent using a computer is out of proportion with benefits (36.6%) and costs of healthcare are likely to increase following computerisation (29.6%).

Although nurses in KNH had a more positive attitude towards computerisation the nurses in AKUH were more knowledgeable on computing (p < 0.001). The mean knowledge score at AKUH was 7.52 out of 9 compared to a mean score of 5.03 among nurses in KNH.

Age of nurses showed a statistically significant association with attitude towards computerisation (p = 0.039). Within KNH nurses aged less than 40 years had significantly higher attitude scores than that of nurses in age groups above 40 years. At AKUH nurses aged above 40 years had the lowest attitude scores (mean = 64.5) followed by those in the youngest age group, 20–25 years (mean = 66.85). Gender did not significantly impact on attitudes towards computerisation (p = 0.86). Professional training had significant impact on the respondents’ attitude towards computerisation (p = 0.025); respondents with University education were more positive toward computerisation. The length of nursing experience did not show a statistically significant association with the attitude of nurses towards computerisation (p = 0.527).

Access to computers was associated with positive attitudes but this association was not statistically significant (p = 0.531). Exposure to computers showed a significant association with attitude towards computerisation (p = 0.025).

The association between access to computers at home and work and attitudes towards computerisation was not statistically significant (p = 0.114).

## Discussion, conclusion and recommendation

According to the results of this study, the nurses have a positive attitude towards computerisation. These findings are consistent with reports from other studies [14,19,24,30]. The findings of this study contradict those of Sultana [25] who found that nurses had negative attitudes towards computerisation. The attitude of nurses towards computers has improved over the years.

The results revealed that both the users and non-users had positive attitudes towards computerisation as found in other studies [20]. However, it was interesting to find nurses in KNH (non-users) had a significantly higher attitude score compared to those in AKUH (users). Apparently, longer exposure to computer use significantly influences attitudes towards computerisation compared to shorter or non-exposure.

Significant association between age and attitudes towards computerisation was found; this mirrors the findings in other studies [12,19]. In this study it was found that nurses aged 40 years and below had higher attitude scores than those aged above 40. This reflects Simpson and Kendrick’s [12] observation that younger nurses were more positively inclined to computer use than their older colleagues. The null hypothesis was therefore rejected. This result contradicts the findings of Getty et al. [20] who found that positive and negative scores were evenly distributed across the age groups. Scarpa et al. [29] too, found no such correlation between age and attitudes. Like in Sultana’s [25] study, it was found that the years spent in the nursing profession did not significantly affect the respondents’ attitudes towards computerisation.

The majority of the respondents in this study were females; a finding also reported by Alquraini et al. [24]. Nursing was and still is largely a female dominated profession. Unlike Alquraini et al’s [24] report, gender in the present study did not have any effect on the nurses’ attitudes towards computerisation. This finding is consistent with those of researchers [30,31] who found no differences between gender and computer attitudes.

Professional qualification influenced the nurses’ attitudes with bachelor degree and higher diploma holders posting high scores compared to certificate holders. This is consistent with other findings [19,24] but contradicted Sultana’s [25].

Nurses with longer durations of exposure to computers (at least three years) at both hospitals were likely to have more positive attitudes than those with relatively shorter durations of computer use. Brumini et al. [19] and Alquraini et al. [24] found a positive correlation between longer use of computers and positive attitudes. These findings support Alquraini et al’s [24] suggestion that a prolonged use of computers enhanced the skill levels of the users, leading to a positive attitude. The findings contradict those of Garland and Noyes [32] who found computer experience was insignificant in predicting positive attitudes towards computers.

A number of positively worded statements received strong endorsement from the respondents. For example, the statements that linked computer use to reduction of paperwork and improved care were greatly supported by the nurses. The respondents also strongly agreed with the idea that computers made nurses work efficient and much easier. This confirms that nurses’ attitudes towards computerisation would be favourable if they were aware of the benefits as found in other studies [33].

In terms of use of information technology, all except one nurse in AKUH (users) had an email account. In KNH 106 of nurses (non-users) indicated that they had an email account. In addition, more AKUH nurses reported that they were capable of preparing and using PowerPoint presentation than their counterparts in KNH. As expected, more nurses in AKUH reported regular use of computers compared to those in KNH. These findings are quite significant given that formal computer training in the institutions that prepare nurses is either limited or if in existence, has not been documented.

### Limitations of the study

Purposive sampling in itself has inherent selection bias; hence generalisation of the results of this study is limited.

## Conclusion

The results of this study reveal that the general attitudes of nurses towards computerization were positive. The findings further disclosed that nurses with little or no experience in using computers in the places of work (non-users) and those from a hospital that had instituted use of computers (users) both had positive attitudes towards computerisation, although the former were more positive.

Nurses were of the opinion that computers are relevant in their field (nursing). They were also aware of the benefits of computers to their professional practice and that seemed to influence their attitudes. Variables such as age, exposure to computer use and level of education have an influence on the nurses’ attitudes towards computerisation. Many respondents had knowledge of basic computer operations and many had e-mail addresses. If any policy maker needed to assess the preparedness of nurses concerning use of computers, this finding would be a good starting point.

### Recommendations

Computer training should be incorporated both in in-service and pre-service nursing curricula or programs.

A nationwide or sector wide study similar to this one is appropriate.

### Lessons learned and impact

The following lessons can be drawn from this study:

Kenyan nurses have a positive attitude towards computerisation

Introduction of computer systems in hospitals is unlikely to attract adverse reaction from the nurses

What is true in one continent may not be true in another.

The impact of the will be established when KNH nurses are evaluated following the recent adoption of computerisation in their hospital.

## Competing interests

The authors declare that they have no competing interests.

## Authors’ contributions

KKM designed the study, collected and analyzed data and drafted the manuscript. KLW, KAK and MM designed the study, critically reviewed and revised the manuscript for important intellectual content. All authors read and approved the final manuscript.

## Pre-publication history

The pre-publication history for this paper can be accessed here:

http://www.biomedcentral.com/1472-6947/14/35/prepub
